# Supplementary material for: Modulation of polar auxin transport identifies the molecular determinants of source–sink carbon relationships and sink strength in poplar
Source: Tree Physiol. 2023 May 20;44(13):82–101. doi: 10.1093/treephys/tpad073 (PMC11898627; doi:10.1093/treephys/tpad073)
Supplement: Sup_Figures_tpad073 [file sup_figures_tpad073.docx]

**Supplementary data**


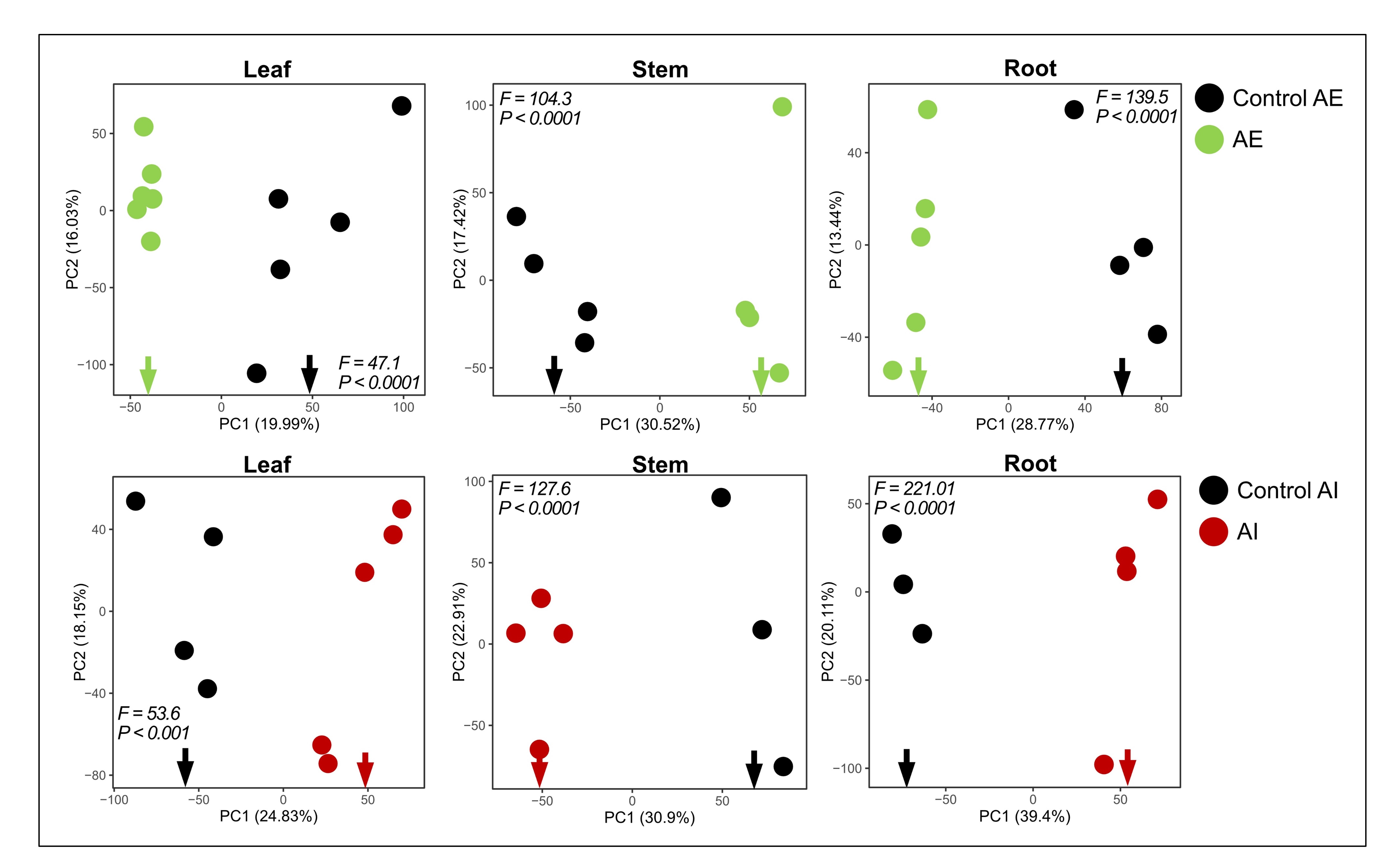


**Figure S1.** Principal Component Analysis (PCA) of total metabolomic profiles from leaf, stem, and root tissues in AE and AI foliar sprayed samples along with their respective control plant tissues.


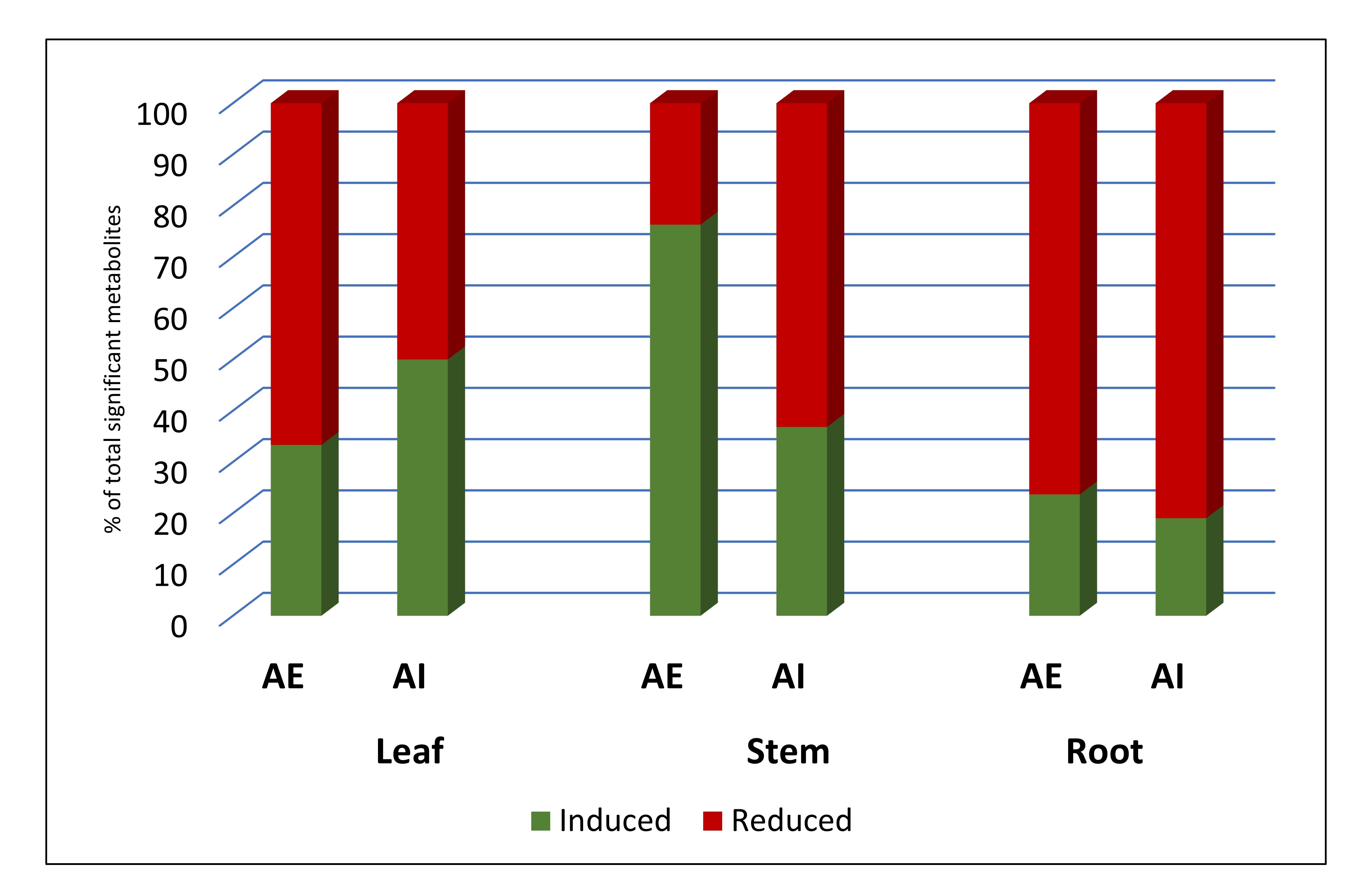


**Figure S2.** The total number of significantly altered metabolites in AE and AI-treated leaf, stem, and root tissues vs. their respective control tissues.


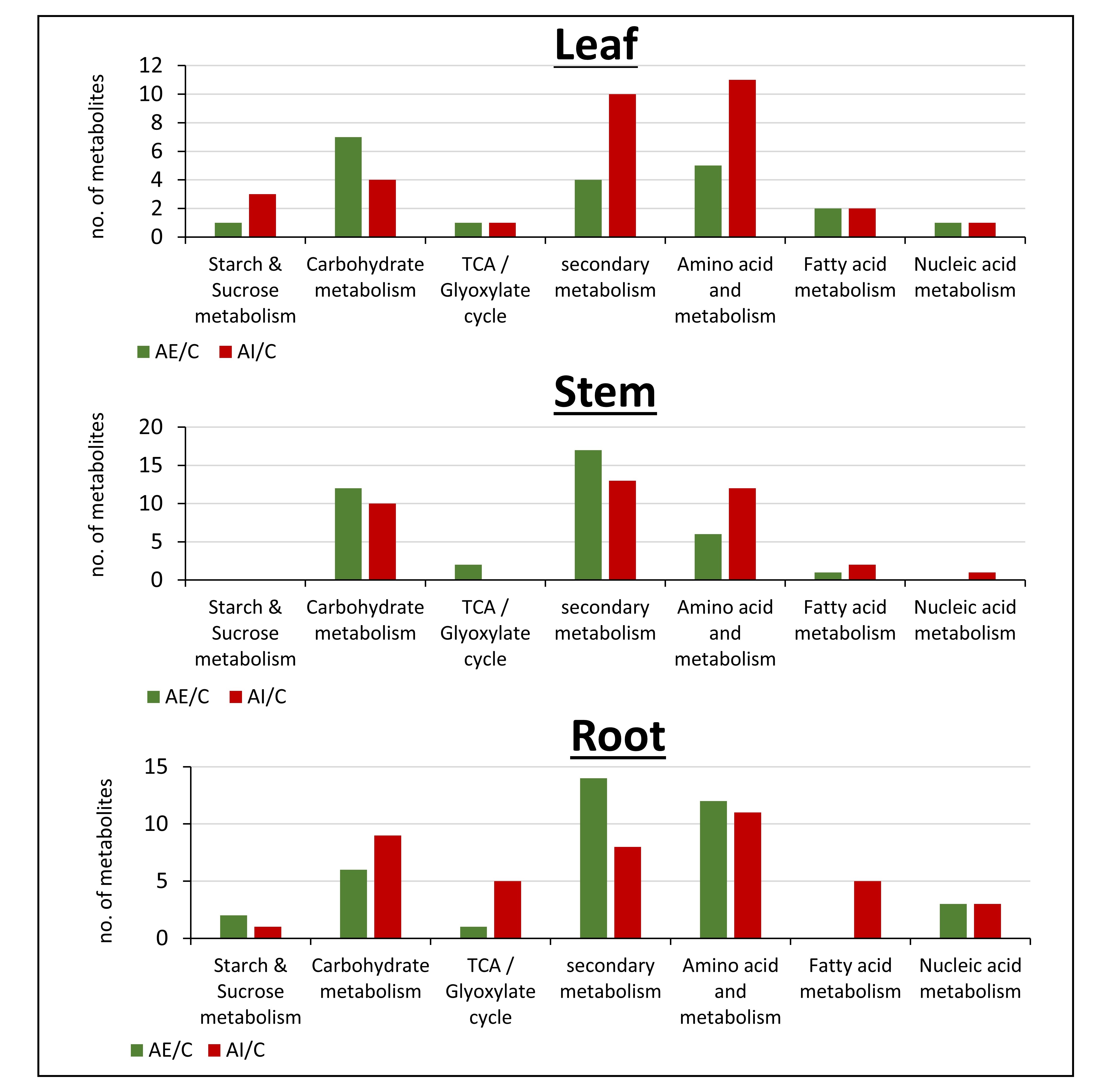


**Figure S3-A.** The total number of significant metabolites in AE and AI-treated leaf, stem, and root tissues vs. control tissues categorized into seven major KEGG pathway categories.


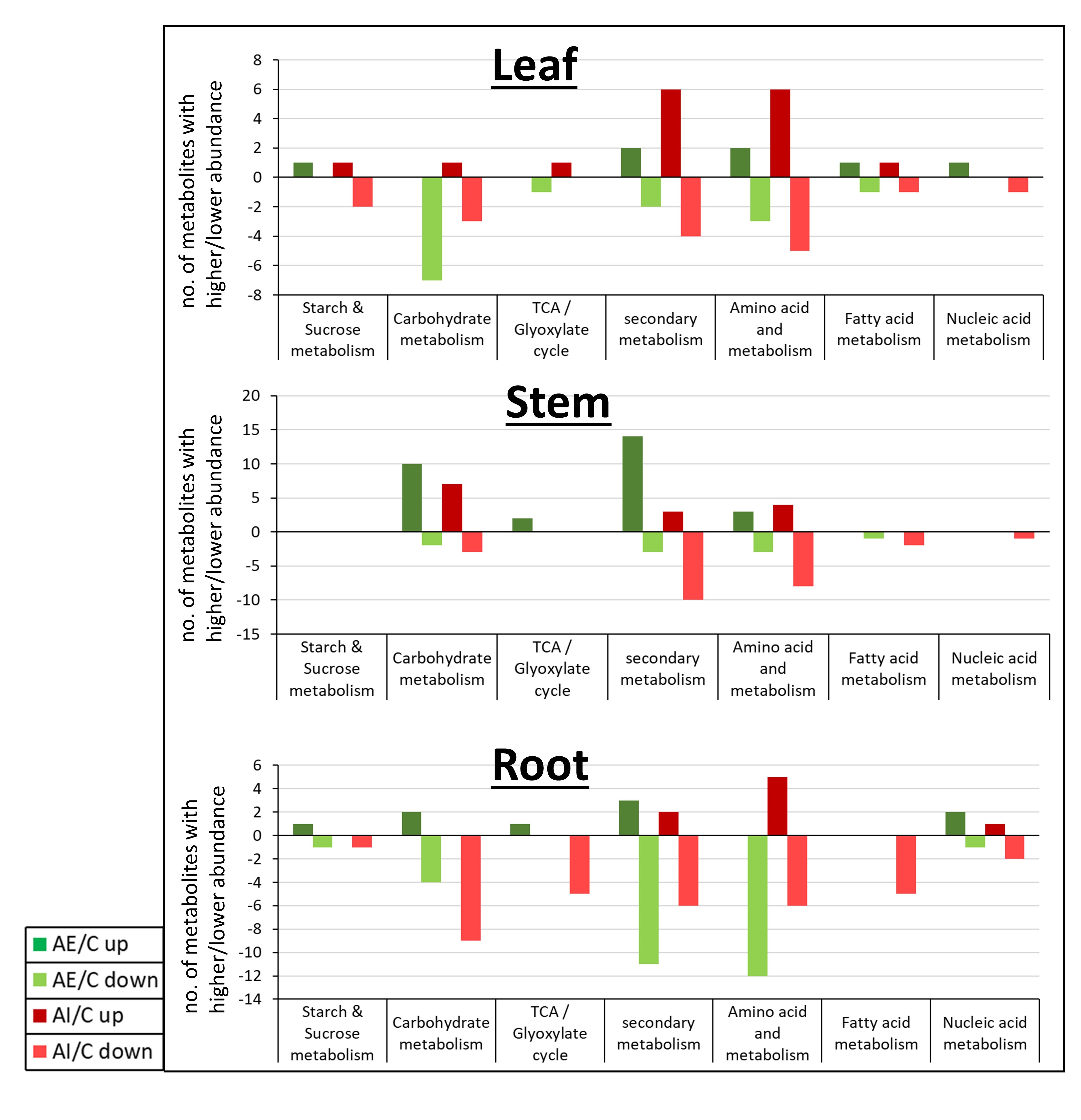


**Figure S3-B.** The total number of significant metabolites with higher and lower abundance levels in AE and AI-treated leaf, stem, and root tissues vs. control categorized into seven major KEGG pathway categories.


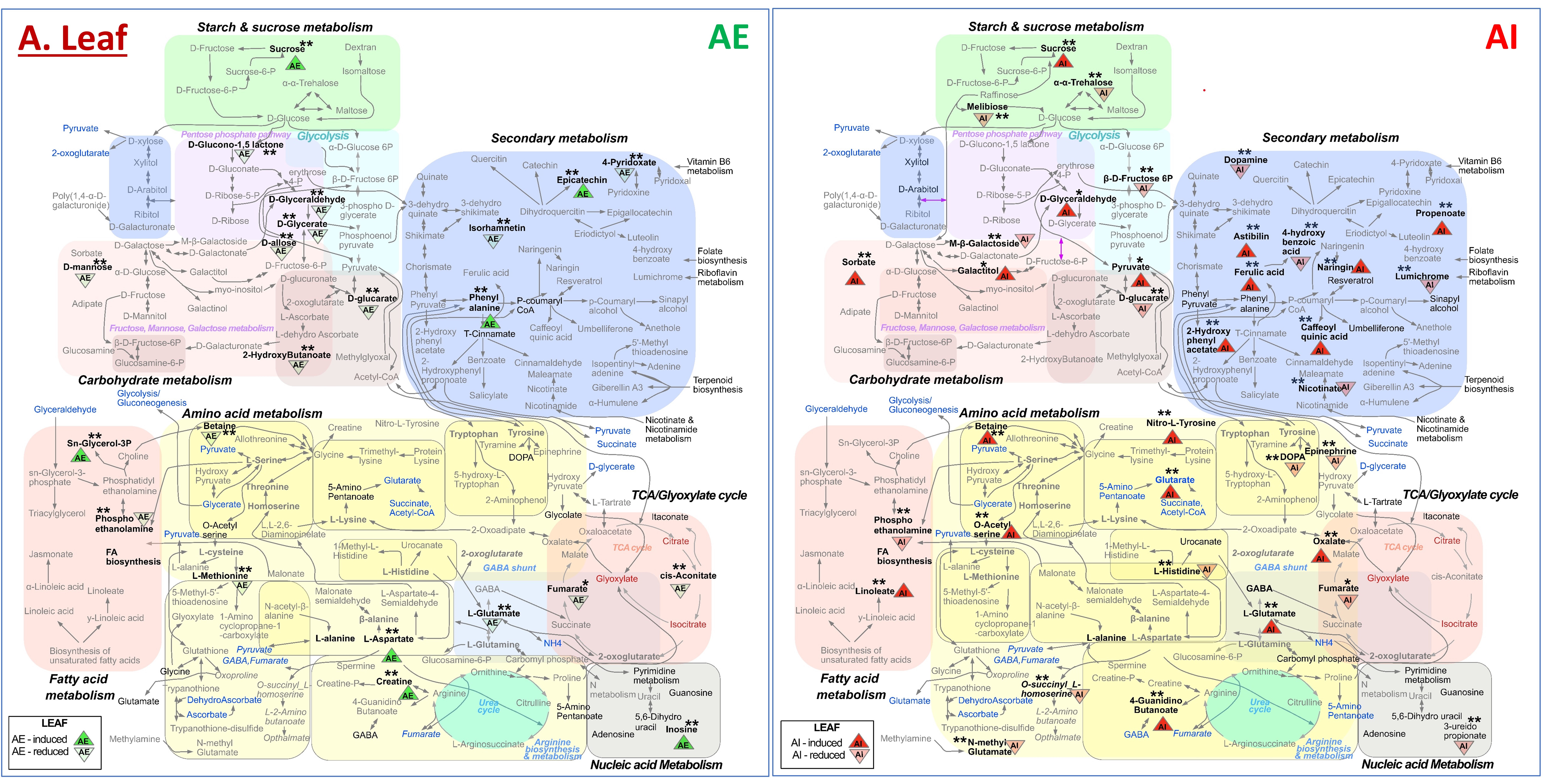


**Figure-S4-A.** Metabolic pathway outline of leaf tissue under AE/AI foliar-sprayed and control conditions.


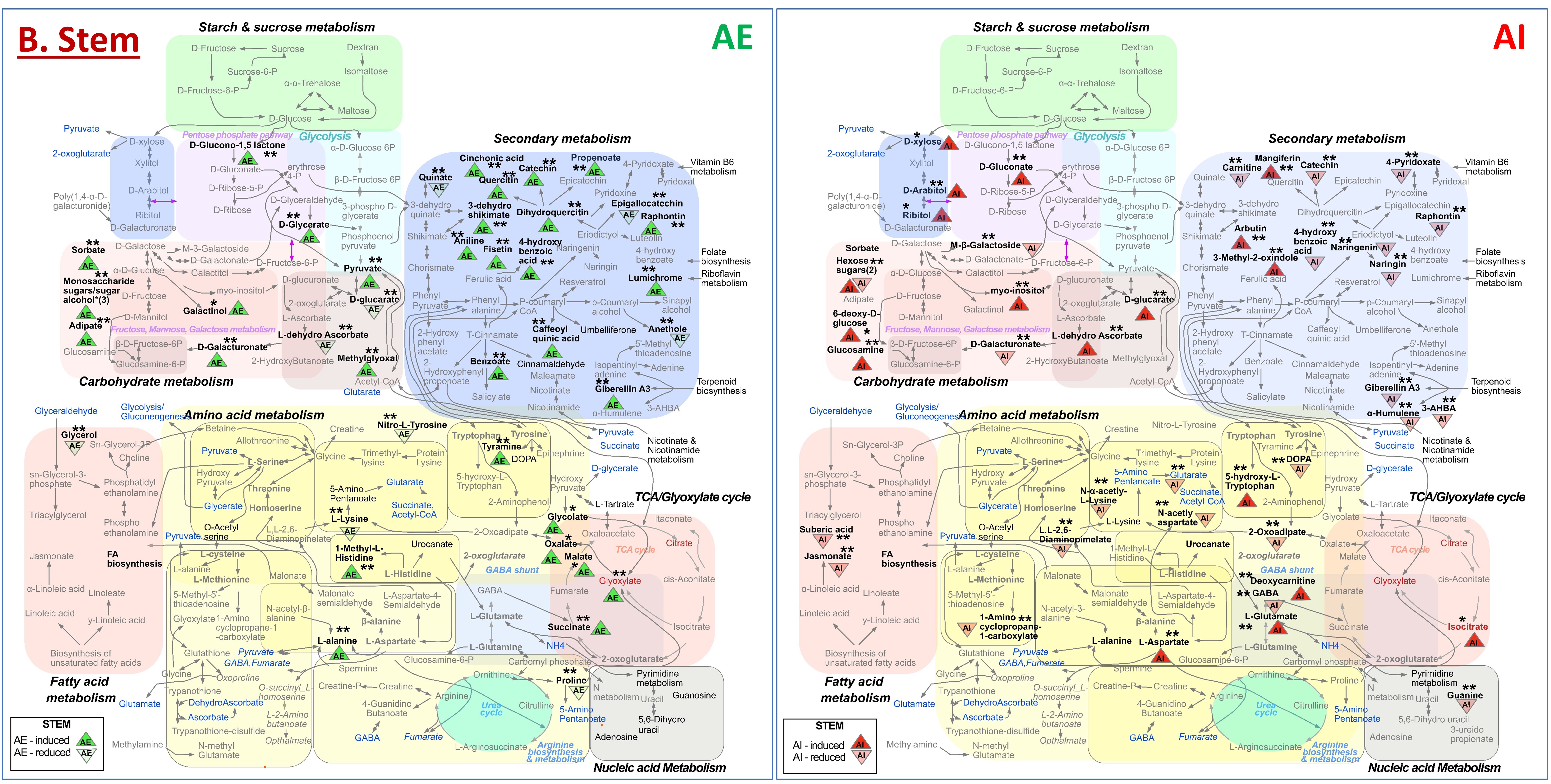


**Figure-S4-B.** Metabolic pathway outline of stem tissue under AE/AI foliar-sprayed and control conditions.


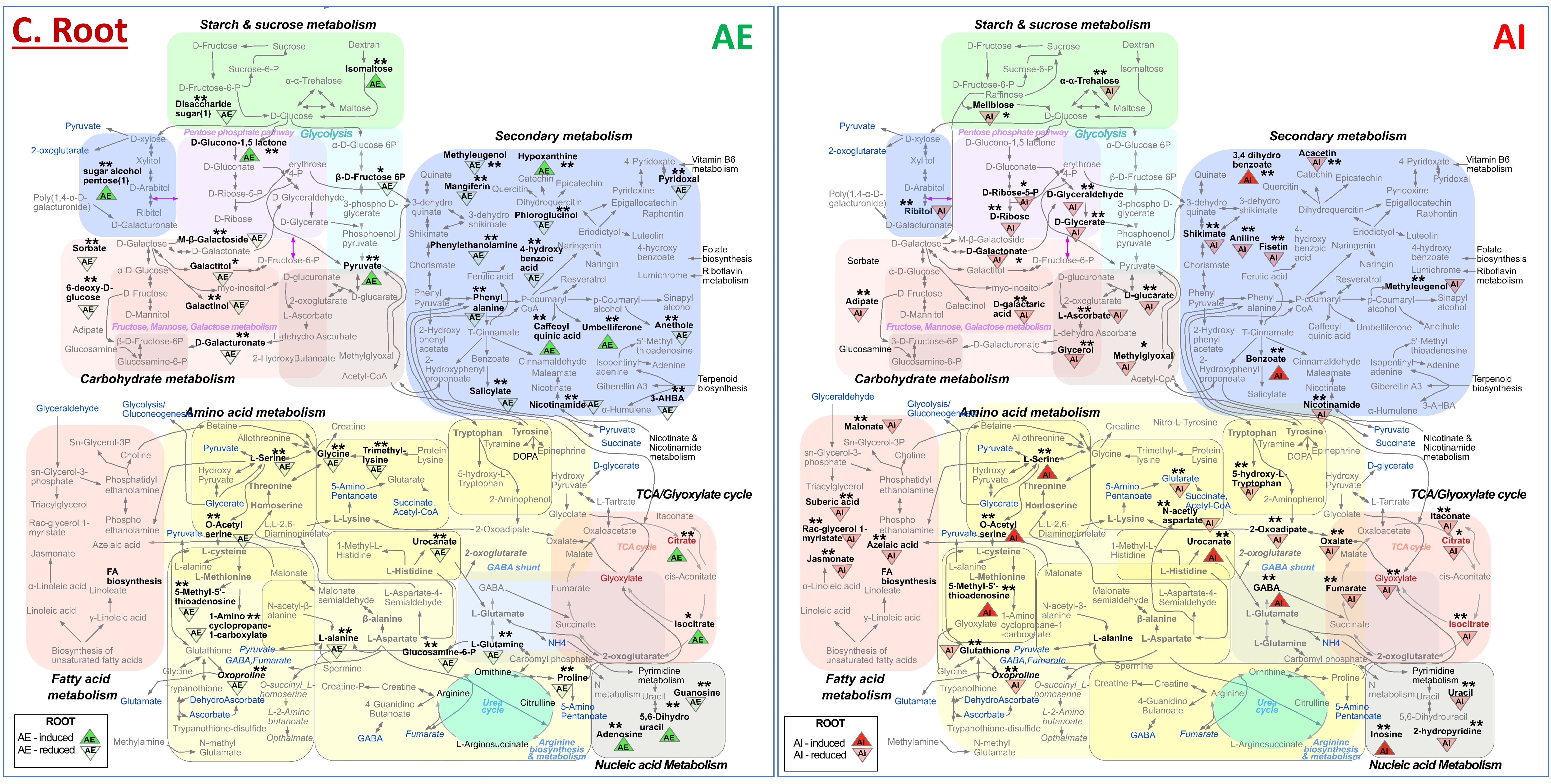


**Figure-S4-C**. Metabolic pathway outline of root tissue under AE/AI foliar-sprayed and control conditions.
